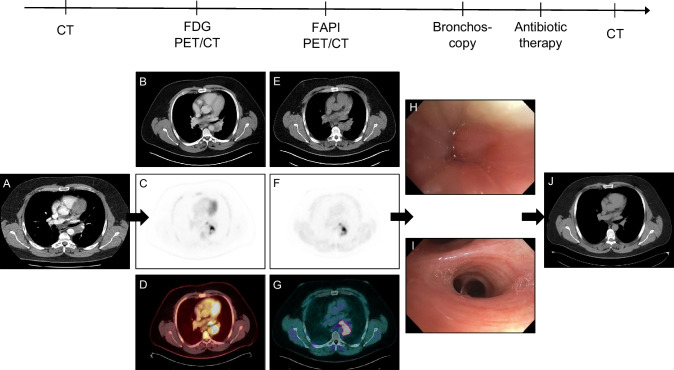# Rabbit fever: granulomatous inflammation by *Francisella tularensis* mimics lung cancer in dual tracer ^18^FDG and ^68^Ga-FAPI PET/CT

**DOI:** 10.1007/s00259-023-06175-7

**Published:** 2023-03-13

**Authors:** Mathias Meetschen, Patrick Sandach, Kaid Darwiche, Dirk Theegarten, Annette Moter, Benedikt Michael Schaarschmidt, Ken Herrmann, Wolfgang P. Fendler, Hubertus Hautzel, Marcel Opitz

**Affiliations:** 1 Institute of Diagnostic and Interventional Radiology and Neuroradiology, University Hospital Essen, University Duisburg-Essen, Essen, Germany; 2 Department of Nuclear Medicine, University of Duisburg-Essen and German Cancer Consortium (DKTK)-University Hospital Essen, Essen, Germany; 3 Department of Pulmonary Medicine, Section of Interventional Pneumology, Ruhrlandklinik, University Hospital Essen, Essen, Germany; 4 Institute of Pathology, West German Cancer Center, University Hospital Essen, University Duisburg-Essen, Essen, Germany; 5 Institute of Microbiology, Infectious Diseases and Immunology, Biofilmcenter Charité-Universitätsmedizin Berlin, Berlin, Germany; 6 Moter Diagnostics, Berlin, Germany

A 39-year-old hunter presented with chills, headache, limb pain, tachycardia, hypertension, ventricular extrasystoles, elevated inflammatory values, and persistent chest pain. A CT scan revealed a mass on the left hilus (A). Due to suspicion of lymphoma or lung cancer 1 week later, an ^18^FDG (B–D) plus a ^68^Ga-labeled fibroblast activation protein inhibitor (^68^Ga-FAPI) PET/CT scan (E–G) were performed. The hilar mass increased in size (B, E) and demonstrated both intense ^18^FDG uptake (SUVmax 24.5) (C, D) and ^68^ Ga-FAPI accumulation (SUVmax 23.2) (F, G) strongly indicating malignancy. However, subsequent EBUS-TBNA and EUS-B yielded necrotizing granulomatosis (H). Finally, a bone-hard mass on the left hilus discharging creamy pus was resected by VATS. Pathological and microbiological workup evidenced *Francisella tularensis* infection by FISHseq analysis (Fluorescence in situ hybridization combined with 16S rRNA gene amplification and sequencing [1]), ELISA, and Western blot. Postoperative bronchoscopy demonstrated re-established bronchus patency (I). After antibiotic therapy with gentamicin and ciprofloxacin, no recurrence was detectable on CT control 20 weeks later (J).

^18^FDG PET/CT is one of the diagnostic mainstays in oncology and standard imaging in patients with lung mass. However, its specificity is impaired due to inflammation-induced uptake. In tularemia, a granulomatous inflammation, ^18^FDG PET/CT revealed an uptake pattern indicative for lung cancer in more than 50% of all cases [2]. ^68^Ga-FAPI emerged as an alternative tracer for tumor imaging, as FAP is expressed in > 90% of epithelial cancers [3, 4]. ^68^Ga-FAPI shows high uptake and tumor-to-background ratio in primary lung cancer and in metastatic lesions of other tumor types located in the lung [5–9]. Although promising data on this new radiotracer are increasing, false-positive results in non-malignant diseases with FAP uptake have been reported [10]. Also, chronic infections like tuberculosis might occasionally demonstrate increased ^68^Ga-FAPI [11]. On the other hand, in a head-to-head comparison (sub)acute inflammation in lymph nodes after COVID-19 vaccination induces no ^68^Ga-FAPI accumulation besides a positive FDG signal [12].

Our case adds tularemia to the scope of potential granulomatous inflammation-induced pitfalls in hybrid imaging coming along with increased high FDG uptake but also high FAP expression. However, given an already known diagnosis of granulomatous disease like tularemia, ^68^Ga-FAPI PET/CT might be suitable to assess extent and activity of chronic inflammation.